# Development of a Bio-Layer Interferometry-Based Protease Assay Using HIV-1 Protease as a Model

**DOI:** 10.3390/v13061183

**Published:** 2021-06-21

**Authors:** Márió Miczi, Ádám Diós, Beáta Bozóki, József Tőzsér, János András Mótyán

**Affiliations:** 1 Department of Biochemistry and Molecular Biology, Faculty of Medicine, University of Debrecen, 4032 Debrecen, Hungary; miczimario@med.unideb.hu (M.M.); bobea86@gmail.com (B.B.); tozser@med.unideb.hu (J.T.); 2 Doctoral School of Molecular Cell and Immune Biology, University of Debrecen, 4032 Debrecen, Hungary; dios.adam@med.unideb.hu; 3 Department of Pediatrics, Faculty of Medicine, University of Debrecen, 4032 Debrecen, Hungary

**Keywords:** protease assay, protease, recombinant fluorescent protein substrate, bio-layer interferometry, human immunodeficiency virus, HIV-1, BLItz

## Abstract

Proteolytic enzymes have great significance in medicine and the pharmaceutical industry and are applied in multiple fields of life sciences. Therefore, cost-efficient, reliable and sensitive real-time monitoring methods are highly desirable to measure protease activity. In this paper, we describe the development of a new experimental approach for investigation of proteolytic enzymes. The method was designed by the combination of recombinant fusion protein substrates and bio-layer interferometry (BLI). The protease (PR) of human immunodeficiency virus type 1 (HIV-1) was applied as model enzyme to set up and test the method. The principle of the assay is that the recombinant protein substrates immobilized to the surface of biosensor are specifically cleaved by the PR, and the substrate processing can be followed by measuring change in the layer thickness by optical measurement. We successfully used this method to detect the HIV-1 PR activity in real time, and the initial rate of the signal decrease was found to be proportional to the enzyme activity. Substrates representing wild-type and modified cleavage sites were designed to study HIV-1 PR’s specificity, and the BLI-based measurements showed differential cleavage efficiency of the substrates, which was proven by enzyme kinetic measurements. We applied this BLI-based assay to experimentally confirm the existence of extended binding sites at the surface of HIV-1 PR. We found the measurements may be performed using lysates of cells expressing the fusion protein, without primary purification of the substrate. The designed BLI-based protease assay is high-throughput-compatible and enables real-time and small-volume measurements, thus providing a new and versatile approach to study proteolytic enzymes.

## 1. Introduction

Proteases (PRs) are among the most extensively studied enzymes due to their critical role in many biological pathways including cell proliferation, differentiation, apoptosis and other physiological processes such as wound healing, blood clotting and food digestion. On the other hand, proteolytic events may be associated with pathological conditions such as cancer, Alzheimer disease, thrombosis and infectious diseases, e.g., the human immunodeficiency virus type 1 (HIV-1) infection. As a consequence of their special features, the proteases are utilized in numerous molecular biological applications [1] and many of them are regarded as crucial targets of drug development. Accordingly, their characterization by efficient and sensitive protease assay methods is essential both in academic and industrial areas.

The recombinant proteins are versatile substrates of protease assays. Numerous fusion protein substrates have been designed to date, and various methods are available for the detection of proteolytic activity. For example, proteolysis-activated fluorescent reporter substrates can be applied in cell culture-based protease assays, which have been reported for HIV-1 protease [2], caspases [3] and for SARS-CoV-2 main protease [4,5], while other types of recombinant proteins were used as substrates to detect the intracellular activity of tobacco etch virus (TEV) protease [6,7]. Besides cell based-assays, various recombinant substrates have been designed for homogenous, separation-based or heterogenous in vitro assays [8,9,10] and for fluorescent resonant energy transfer (FRET) [11] and bioluminescent resonant energy transfer (BRET)-based [12] measurements, as well.

Our research group has designed a recombinant fluorescent protein (RFP)-based protease assay which is based on the use of such substrates that contain N-terminal hexahistidine (His_6_) and maltose binding protein (MBP) tags, followed by the cleavage site sequence of the studied PR and a fluorescent protein (FP) tag [13,14,15]. These His_6_-MBP-FP substrates can be used in a separation-based assay where the substrates are coated onto the surfaces of nickel nitrilotriacetic acid (Ni-NTA) magnetic beads. Upon the processing of the immobilized substrates, the product formation can be followed by either fluorimetry or by polyacrilamide gel electrophoresis (PAGE), using native or denaturing conditions. Both native and denaturing PAGE may be utilized for the analysis of reactions if the non-immobilized substrates are digested in the solution (homogenous assay). Either conventional Coomassie-staining or UV/blue-light transillumination can be used to visualize the proteins in the gel; the latter detection can be applied in the case of native PAGE or if the denatured proteins are partially renatured in the gel after denaturing PAGE [13]. Western-blot may be also used for fusion tag-specific detection (e.g., using anti-MBP antibody) [16]. The magnetic bead-based protease assay can be performed both in microcentrifuge-tubes [17,18] and in 96-well microtiter plates [15,19]. Purified enzymes are used in the conventional setup, but cleavage reactions may be performed in total cell lysates [16], as well. Although the designed His_6_-MBP-FP substrates can be applied by using multiple experimental settings, none of the applications that have been tested to date enabled real-time detection of product formation.

The bio-layer interferometry (BLI) is a widely applied method for the determination of biomolecular interactions. To study protein–protein interactions, a bait molecule can be immobilized to a biosensor’s surface. The interaction partner of the immobilized bait molecule (analyte) can associate with the biomolecular layer, which can be monitored by detecting the interference pattern of white light (the signal is generated by the shift of the wavelength of the reflected light, in nm/s) which is changed as a function of the bio-layer’s thickness on an optical biosensor [20]. A main advantage of BLI is the continuous detection that enables real-time monitoring of light interference whose change directly correlates with the changes of the surface’s thickness caused by the association of the analyte.

Although a wide variety of intermolecular interactions can be investigated by BLI, this method is not applied widely to measure enzyme activity. To date, only few studies have been reported about the investigation of enzyme activity by BLI. As an example, Kojima et al. studied the activity of horseradish peroxidase (HRP) in a real-time BLI-based assay, using an immobilized enzyme [21]. Most commonly, PRs are used in BLI assays to enzymatically remove remaining molecules from sensor surfaces [22], but to our knowledge, the application of BLI in protease assays has not been published to date. Surface plasmon resonance (SPR) was shown to be suitable for the detection of protease activity via recombinant substrate-based technology [23,24], but in contrast to BLI, this method is not considered to be fully compatible with high-throughput screening (HTS).

In this work, our aim was to apply versatile recombinant fluorescent substrates and design a BLI-based real-time protease assay. We describe the optimization of the conditions for RFP immobilization and proteolytic cleavage reactions, and the application of the designed experimental procedure to determine relative cleavage efficiencies of HIV-1 PR using His_6_-MBP-FP substrates containing different cleavage site sequences.

The HIV-1 PR plays a crucial role in the life-cycle of HIV-1 by the cleavage of the viral Gag and Gag-Pro-Pol polyproteins which are translated from the non-spliced genomic RNA. The matrix (MA), capsid (CA) and nucleocapsid (NC) major structural proteins are expressed from the *gag*, the *pol* codes for the viral enzymes reverse transcriptase (RT), integrase (IN) and PR. The role of the mature homodimeric PR is the limited proteolysis of the polyproteins into functional subunits, and the intradomain bonds are cleaved at specific sites with different cleavage rates. The function of HIV-1 PR is essential for the viral infectivity; therefore, the treatment of acquired immunodeficiency syndrome (AIDS) includes the use of inhibitors that block its activity [25,26].

This paper is dedicated to the loving memory of Dr. Stephen Oroszlan, who made an essential contribution to retrovirology, including the field of HIV protease and other retroviral proteases [27,28,29]. Our research group, the Laboratory of Retroviral Biochemistry was established at the University of Debrecen in 1992 by József Tőzsér, after working together with Dr. Oroszlan in the Molecular Virology and Carcinogenesis Laboratory at NCI-Frederick Center for Cancer Research. Most studies of our research group followed his ideas, including the investigation of HIV-1 and other retroviral proteases, antiviral inhibitors and enzyme specificity, works which have been covered by reviews [25,27,30]. Several studies of our research group—including the present work—were inspired by the specificity studies of our laboratory performed previously together with Dr. Oroszlan and his colleagues.

## 2. Materials and Methods

All materials were obtained from Sigma-Aldrich (St. Louis, MI, USA), otherwise it is indicated.

### 2.1. Expression and Purification of HIV-1 PR

The plasmid (pET11a) encoding HIV-1 PR with five stabilizing mutations (Q7K, L33I, L63I, C67A and C95A) was kindly provided by Dr. John M. Louis (Laboratory of Chemical Physics, NIDDK, NIH); the construction of the plasmid was described previously [31]. This enzyme was considered as wild-type (HIV-1 PR_wt_). For the expression of HIV-1 PR_wt_, *Escherichia coli* BL21(DE3) cells transformed with the expression plasmid were grown in Luria-Bertani (LB) medium (supplemented with 100 µg/mL ampicillin) at 37 °C to at least an absorbance of 0.6–0.8 at 600 nm. Then, 1 mM isopropyl β-D-1-thiogalactopyranoside (IPTG) was added to the cells to initiate the protein expression, followed by a 3 h incubation. Cells were pelleted by centrifugation at 6,000 *g* for 20 min at 4 °C and the pellet was treated as described previously [32]. Pellet was suspended in 20 volume of extraction buffer A (50 mM Tris, 10 mM EDTA, pH 8) and lysed by ultrasonication on ice in the presence of 0.1 mg/mL lysozyme. The lysate was centrifuged at 20,000 *g* for 20 min at 4 °C, suspended in extraction buffer B (50 mM Tris, 10 mM EDTA, 1 M urea, 1% Triton X-100, pH 8) and centrifuged again. Pelleted inclusion bodies were suspended in extraction buffer C (50 mM Tris, 5 mM EDTA, 7.5 M guanidine-HCl, pH 8.0) and after centrifugation, the supernatant was collected and filtered through a 0.22 µm pore size filter (Merck-Millipore, Burlington, MA, USA) and diluted in a 1:1 ratio with distilled water before applied on a C18 column to RP-HPLC. Fractions were collected at a concentration of 40% acetonitrile containing 0.05% TFA. Fractions were analyzed by SDS-PAGE to select the fractions of highest purity (>90%). The protein was folded by dialysis into 0.05 M formic acid at pH 2.5 followed by a dialysis using acetate buffer (50 mM sodium-acetate, pH 5). Protein concentration was determined by NanoDrop 2000 instrument (Thermo Fisher Scientific, Waltham, MA, USA) at 280 nm.

The truncated form of HIV-1 PR (HIV-1 PR_ΔNΔC_ lacking 1–4 N- and 96–99 C-terminal residues) was expressed, purified and dialyzed using the conditions applied for HIV-1 PR_wt_; the HIV-1 PR_ΔNΔC_ expression plasmid [33] was also kindly provided by Dr. John M. Louis.

The preparation of trypsin was performed based on the methods described previously [34,35,36] and was a kind gift of Dr. András Szabó.

### 2.2. Cloning, Expression and Purification of Recombinant Fluorescent Substrates

The cloning, expression and purification of RFP substrates were performed based on the method described previously [13,14,15], but using a slightly modified lysis buffer (100 mM NaCl, 50 mM Tris-HCl, pH 7.5). The oligonucleotide primers coding for the HIV-1 MA/CA cleavage site sequences are shown in Table 1. The success of cloning was confirmed by sequencing.

### 2.3. Preparation and Investigation of Ni^2+^-, Cu^2+^-, Zn^2+^- and Co^2+^-Charged NTA Biosensors

The Ni-NTA biosensors (Pall Forté Bio, Fremont, CA, USA) were soaked in hydration buffer (50 mM tris HCl, 0.5 mM EDTA, 1 mM dithiothreitol (DTT), 1% glycerol, pH 8) for at least 10 min prior to use, followed by a washing step with EDTA solution (250 mM EDTA, pH 8). Afterwards, each sensor was dipped into the solution of a divalent cation (10 mM NiSO_4_, 10 mM ZnCl_2_, 10 mM CoCl_2_ or 10 mM CuCl_2_) in two cycles, each for 60 s. Finally, excess of cation was washed out from the sensors using assay buffer containing assay buffer A (50 mM sodium acetate, pH 5) and assay buffer B (0.275 M di-sodium hydrogen phosphate, 0.2 M sodium di-hydrogen phosphate, 4 M NaCl, 10% glycerol, pH 5.6) in a 1:1 ratio. The baseline was established in assay buffer for 30 s, then the association phase was examined in assay buffer containing His_6_-MBP-VSQNY*PIVQ-mEYFP substrate in 0.7 µM final concentration for 120 s in 4 µL volume, followed by the dissociation phase in assay buffer for 120 s in 250 µL volume.

The Ni-NTA biosensors were regenerated at low pH using phosphoric acid (pH 2.5), based on the manufacturer’s instructions.

Data were evaluated using BLItz Pro 1.2 software and fitted to 1:1 binding model with local fitting (Langmuir).

### 2.4. Real-Time HIV-1 PR Activity Measurements by BLI Using Purified Substrates

The Ni-NTA sensors were soaked in hydration buffer prior to run for 10 min. At the beginning of the assay, we used a 30 s baseline step in assay buffer (tube-mode, 250 µL volume) followed by the loading of substrate (14 µM) for 60 s (drop-mode, 4 µL volume). Excess substrate and non-specifically bound molecules were washed out from the sensors for 60 s using assay buffer (tube-mode). A final washing step was performed using assay buffer for 30 s (drop-mode) in order to minimize baseline-shift from tube-mode to drop-mode transition (drop-mode). HIV-1 PR_wt_ was diluted with assay buffer B in 1:1 ratio in advance of use, further dilutions were prepared with assay buffer. Each enzymatic reaction was run in a 4 µL volume (drop-mode). The same conditions were applied if HIV-1 PR_ΔNΔC_ was used.

For the inhibitory experiments, the conditions were slightly modified due to the presence of DMSO in the reactions. An extra washing step was included, using 3.8 uL of reaction buffer supplemented with 0.2 µL DMSO. The final concentration of active HIV-1 PR_wt_ was 9 nM and atazanavir was applied in a final concentration of 2 nM and 2 µM. Atazanavir was obtained through the NIH AIDS Reagent Program, Division of AIDS, NIAID, NIH (Germantown, MD, USA) [37]. Control experiment contained only 0.2 µL DMSO with HIV-1 PR_wt_ diluted in assay buffer. Blank reaction contained assay buffer containing 0.2 µL DMSO. All experiments were run on BLItz instrument (Pall Forté Bio, Fremont, CA, USA) at room temperature with agitation (2200 rpm). Raw data were exported from the BLItz Pro 1.2. software and further analyzed using Microsoft Excel.

### 2.5. BLI Protease Assay Using Non-Purified Substrate

The transformation of BL21(DE3) cells, the protein expression and lysis of cells were performed as described previously [13]. After lysis, the cell suspensions were centrifuged at 10,000 *g* for 20 min, and the supernatants (cell lysates) containing the RFP substrate were collected. The lysates were used for BLI measurements and were analyzed by SDS-PAGE, as well, as it is described below.

In the BLI measurements, a 20 s baseline step was applied using lysis buffer (100 mM NaCl, 50 mM Tris-HCl, pH 7.5), followed by loading of lysates for 30 s. The sensors were washed for 30 s with lysis buffer and then with assay buffer (both in tube-modes). A final washing step was performed in drop-mode (for 30 s) in order to minimize baseline shift between tube-to-drop transition. For proteolysis, HIV-1 PR_wt_ was used.

### 2.6. PAGE Analysis and Densitometry

Electrophoresis of samples was performed using 14% SDS polyacrylamide gel. Before electrophoresis, the samples were supplemented with 6X loading buffer. In the case of denaturing SDS-PAGE, the samples supplemented with the loading buffer (300 mM Tris, 20% glycerol, 0.05% bromophenol blue, 12% SDS, 100 mM β-mercaptoethanol, pH 6.8) were heated at 95 °C for 10 min, then loaded onto the gel, while for native PAGE, the loading buffer was absent from denaturing and reducing agents and no heat treatment was applied. The electrophoreses were run on 120 V in 1X SDS running buffer (0.025 M Tris, 0.192 M glycine, 0.1% SDS, pH 8.5). After denaturing SDS-PAGE, the denatured fluorescent proteins were partially renatured in the gel using the previously described protocol [13,14,15]. To visualize the proteins in the gels, we used conventional Coomassie staining, and UV or blue-light transillumination was also applied.

The intensities of protein bands were determined by the GelAnalyzer 19.1 free desktop application (http://www.gelanalyzer.com; by István Lázár Jr. and István Lázár Sr.) (Date of last accession: 05 April 2021).

### 2.7. Enzyme Kinetic Measurements

The determination of enzyme kinetic parameters was performed using His_6_-MBP-mApple substrates representing wild-type and modified HIV-1 MA/CA cleavage sites (wt-24res and mut-24res), based on the protocols described previously [13,14,15].

The active site titration of HIV-1 PR_wt_ was performed using an HPLC-based method as described previously [13]. For the determination of the active enzyme concentration (22 nM), the oligopeptide substrate representing the wild-type HIV-1 MA/CA cleavage site (VSQNYPIVQ) was applied at 0.47 mM final concentration in 1X assay buffer B, using amprenavir as a potent inhibitor (final concentration ranging from 0.1 nM to 20 nM).

## 3. Results

### 3.1. Recombinants Substrates

**Figure 1:**
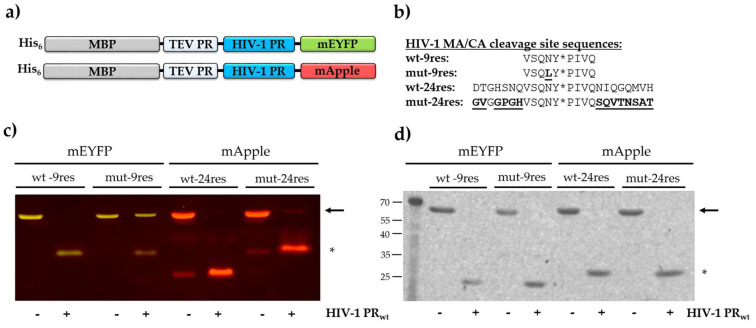
The RFPs as substrates of HIV-1 PR. (**a**) Schematic representation of the RFPs. The substrates contain cleavage sites of tobacco etch virus (TEV) and HIV-1 PR. (**b**) Cleavage site sequences of HIV-1 PR representing wild-type and modified HIV-1 MA/CA cleavage sites are shown, the modified residues are underlined. Asterisks show the cleavage position. (**c**) Native PAGE analysis of uncleaved substrates (~72 kDa) and cleavage products (~26 kDa) after proteolysis by HIV-1 PR_wt_. The proteins were visualized in the gel using blue-light transillumination. (**d**) Reducing SDS-PAGE analysis of uncleaved substrates and cleavage products after proteolysis by HIV-1 PR_wt_. The SDS-PAGE was followed by in-gel renaturation of the separated proteins, which were then visualized using UV transillumination. The substrates and fluorescent tag-containing products are indicated by arrows and asterisks, respectively.

The RFP substrates used for protease assays were prepared based on the previously applied methodology [13,14,15]. The His_6_-MBP-mEYFP (mEYFP, monomeric enhanced yellow florescent protein) and His_6_-MBP-mApple recombinant proteins (Figure 1a) were designed to contain a 9- or a 24-residue-long sequence (9res and 24res, respectively) representing a wild-type (wt) or a modified (mut) natural MA/CA cleavage site of HIV-1 PR. The modified cleavage site sequences were prepared by the substitution of the P2 cleavage site residue (P2-Leu), or by changing the P12-P6 and P5′-P12′ residues of HIV-1 MA/CA cleavage site to those of HIV-1 capsid/p2 (Figure 1b). The protein substrates were expressed in BL21(DE3) *Escherichia coli* cells and purified with immobilized metal affinity chromatography (IMAC) using Ni-NTA magnetic agarose beads.

The rationale behind substrate selection was that the substrate contains a specific cleavage site of HIV-1 PR, and the same RFPs can be applied in other in vitro analyses, as well (gel-based analysis, fluorometric assay). To test whether the purified proteins can be used as substrates in protease assays, cleavage reactions were performed with HIV-1 PR containing five stabilizing mutations [31]; this enzyme was considered as a wild-type enzyme (HIV-1 PR_wt_). The reaction mixtures and the substrate controls were separated by native PAGE and the fluorescent proteins were visualized in the gel by blue-light transillumination (Figure 1c). In agreement with our previous results [13], only the bands of the uncleavaed substrates and the C-terminal cleavage products—containing the fluorescent tag—were detected in the gel, indicating cleavage of substrates at the incorporated MA/CA cleavage site. In the case of wt- and mut-24res substrates, we detected fluorescent bands in substrate controls at molecular weights similar—but not identical—to cleavage products (Figure 1c), as was detected previously in the case of uncleaved mApple-fused wt-9res substrate, as well [14]. The mApple-containing RFPs were found to exhibit lower ability for renaturation as compared to mTurquoise2 proteins [13]; therefore, the apparent products bands appear possibly due to relatively lower stability of mApple-fused substrates. The C-terminal cleavage fragment appeared to have apparently lower molecular weight in the case of wt-24res substrates based on native PAGE, due to different polarity of the wt-24res and mut-24res cleavage site sequences. Such differences were not observed, and the proteins were detected at the expected molecular weight if they were separated using reducing conditions in sodium-dodecyl-sulphate-PAGE (SDS-PAGE) and then visualized by UV transillumination (Figure 1d) or by Coomassie staining (Figure S1). In summary, our results proved that the designed substrates are processed by HIV-1 PR_wt_ and are suitable candidates for the development of the BLI-based PR assay.

### 3.2. Design of the BLI-Based Protease Assay

We chose HIV-1 PR as the model enzyme for the development of the BLI-based protease assay. The His_6_-MBP-mEYFP recombinant protein representing the wild-type HIV-1 MA/CA cleavage site (VSQNY*PIVQ) has already been applied successfully to study HIV-1 PR_wt_ [13,14,15]; therefore, this substrate was used for method development.

Biosensors that are pre-coated with anti-His antibody are available to capture His-tagged proteins, but to avoid unwanted proteolysis of the antibodies, we decided to capture the RFPs using biosensors with immobilized Tris-NTA moieties on their surface.

**Figure 2:**
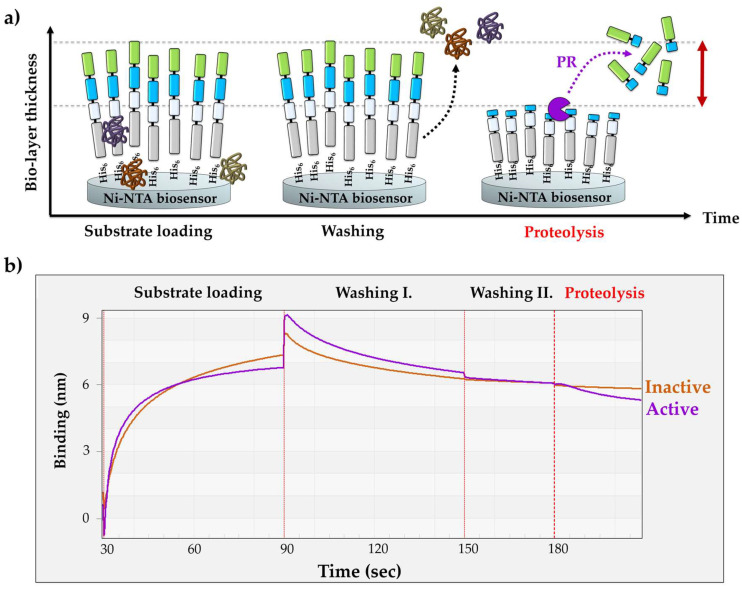
Principle of the BLI-based protease assay. (**a**) Schematic representation of the biosensors during the assay. (**b**) A representative sensogram shows the steps of the assay procedure. After setting up the baseline using Ni-NTA biosensors in tube-mode, the RFP substrates are loaded onto the sensors in drop-mode until saturation. Loading is subsequently followed by tube- and drop-mode washing steps. Cleavage reactions were performed using a catalytically active (HIV-1 PR_wt_, purple) and inactive HIV-1 PR (HIV-1 PR_ΔN__ΔC_, orange) enzyme. The same Ni-NTA biosensor was used for the parallel experiments, and the sensor was regenerated between the measurements at low pH (based on manufacturer’s instructions).

The BLI-based PR assay consists of three main steps (Figure 2a). (i) The first step is the loading step to immobilize the substrates onto the biosensor surface. (ii) The loading is followed by washing steps in order to wash the excess substrates and non-specifically bound proteins from the surface. (iii) The third step is the proteolysis; in this step, the cleavage reaction is initiated by merging the biosensor into the enzyme solution.

For substrate loading, the biosensors were merged into the solution of purified RFP in order to ensure saturation of the surface. The affinity binding of the RFPs to the biosensor surface is enabled by their N-terminal His_6_-tags. The enzyme reactions were performed at high salt concentration and at pH 5.5, close to the pH optimum of HIV-1 PR [38]. The sensors were washed in two steps: (i) the first washing step was performed in tube-mode (the sensor was dip into a microcentrifuge tube containing the working solution) (Washing step I in Figure 2b), while (ii) drop-mode was used for the second step which decreased the applied volume from 250 μL to 4 μL (Washing step II in Figure 2b). In drop-mode, the small-volume working solution was applied to the drop-holder of the BLItz instrument. In tube-mode, there was a moderate dissociation of the substrate, which was completely abolished in the second washing step due to the dissociation-limiting effect of the drop-mode that improved immobilization in the applied conditions. The spontaneous dissociation of the RFSs from the metal affinity surface is dependent on pH and may increase by lowering the pH (<6.0) [13]. The enzyme reactions were performed at pH ~5.5, which may be slightly suboptimal for substrate immobilization, but it was optimal for HIV-1 PR. Working at higher pH, e.g., in case of enzymes having higher pH optimum, may enable more stable interaction between the RFP and biosensor, but testing multiple enzymes was out of the scope of this study.

The washing steps were followed by the proteolysis. The applied buffer conditions of the cleavage reactions were identical with those of the drop-mode washing steps. The enzyme reactions were initiated by merging the sensor into the enzyme solution. Processing of the RFPs by HIV-1 PR_wt_ resulted in a decrease in the bio-layer thickness, causing an intensive decrease in the detected signal (Figure 2b).

Upon the cleavage of the immobilized His_6_-MBP-VSQNY*PIVQ-mEYFP substrate (72.24 kDa) by HIV-1 PR_wt_, the N-terminal cleavage fragment (His_6_-MBP-VSQNY, 44.45 kDa) remained associated with the biosensor, while the C-terminal product (PIVQ-mEYFP, 27.64 kDa) containing the fluorescent tag was released into the solvent. In order to prove that the observed decrease in the signal is caused by the proteolytic digestion of the RFP substrate rather than by substrate dissociation, the experiments were repeated using a mutant HIV-1 PR containing truncated termini. This shortened enzyme (HIV-1 PR_ΔNΔC_) was designed to contain the deletion of the 1–4 and the 96–99 terminal residues [39]. The deleted residues constitute a part of the dimer interface of HIV-1 PR, which is essential for the formation a functional homodimer, not only in case of HIV-1 PR but also in case of several other retroviral proteases [40]. This truncation of the N- and C-termini of HIV-1 PR was found previously to cause enzyme inactivation [39]. Accordingly, we observed only negligible change in the signal using HIV-1 PR_ΔNΔC_, in contrast to the substantial change that was detected for the catalytically active HIV-1 PR_wt_. These results proved that the significant decrease in the BLI signal was caused by the enzymatic processing of RFP substrates and not by spontaneous substrate dissociation.

The His_6_-MBP-VSQNY*PIVQ-mEYFP substrate was found to be processed by trypsin, as well (Figure S2). We found that the signal curve had higher slope upon tryptic digestion as compared to HIV-1 PR, presumably due to a relatively higher change in the layer thickness upon proteolysis because trypsin has multiple cleavage sites in the substrate and can cleave it prior to HIV-1 PR cleavage site, as well. However, the observed differences are not fully comparable if enzymes with different active enzyme concentration, specificity and catalytic properties are used. The results indicate that such substrates may also be applied, the processing of which results in a higher change in the molecular mass of the immobilized substrate, potentially increasing the sensitivity of the method.

Our result implied that the RFP substrates are suitable candidates to set up optimal conditions for real-time enzymatic activity measurement using BLI.

### 3.3. Optimizing Substrate Immobilization on Biosensors

**Figure 3:**
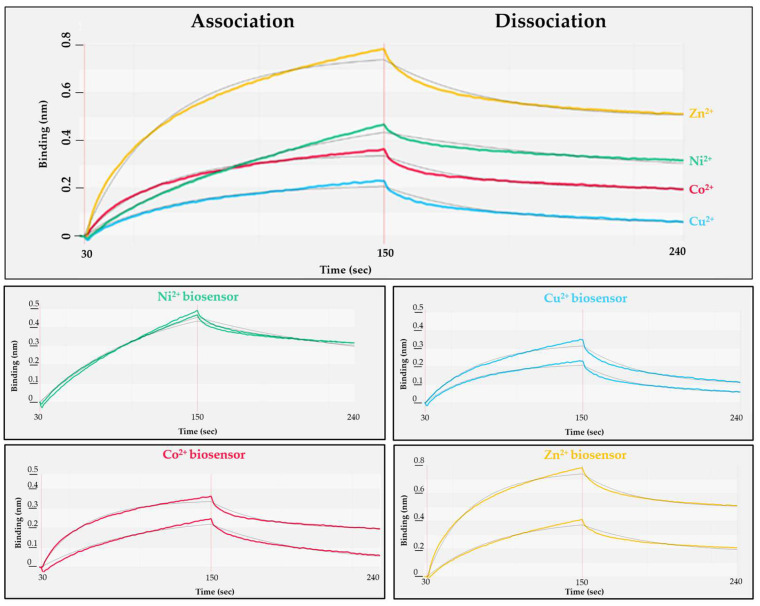
Study of the interaction of different divalent cations with His_6_-MBP-VSQNY*PIVQ-mEYFP substrate. The kinetic parameters determined for the cations are shown in Table 2. Results of two experiments are represented for each cation. The grey lines show fitting of data to 1:1 binding model by BLItz Pro software.

The polyhistidine sequence is known to exhibit strong binding to nickel. The NTA affinity surfaces charged with other divalent cations are also known to be suitable for the immobilization of His-tagged proteins in IMAC [41,42], but there is limited information about their binding kinetics to biosensors. The spontaneous dissociation of RFPs from the biosensor can be avoided by using such cations, which provide relatively small dissociation constant (kd) via sufficient interactions. Additionally, the setup of the conditions that enable regeneration of biosensors (and thereby reproducible binding curves) was also considered to be important when optimal assay conditions are to be determined. We have tested Zn^2+^, Ni^2+^, Co^2+^ and Cu^2+^ divalent cations for RFP immobilization (Figure 3).

The NTA-based biosensors were charged individually with each cation, followed by loading of His_6_-MBP-VSQNY*PIVQ-mEYFP substrate onto the sensor (association phase, Figure 3). After changing the solution to buffer lacking the substrate (dissociation phase, Figure 3), the binding affinity was determined for each cation-charged surface (Table 2).

The most stable interaction was detected between the His_6_-tagged substrate and nickel ions of the tested cations (Table 2, and the Ni-NTA sensor showed slower saturation (Figure 3). Besides binding affinity, the effect of biosensor regeneration on RFP immobilization was also tested, because the regeneration of biosensors (re-charge of the sensors with ions) was considered to potentially affect the reproducibility of protein binding. R_equilibrium_ (the response at steady state) is highly dependent on the analyte concentration (c), association (ka, rate of complex formation) and dissociation (kd, stability of complex formed) rate constant and the maximum binding capacity (R_max_) of the biosensor surface as the following equation demonstrates: R_equilibrium_ = ((ka*c)/(ka*c + kd))*R_max_. Improper regeneration (residual protein on biosensor, insufficient ion replacement) or damaging the sensors may lead to decreased R_max_, thus to a lower R_equilibrium_. We found that with the exception of Ni-NTA surface, the biosensors charged with the other cations showed relatively lower efficiency of sensor regeneration and the R_equilibrium_ values were less reproducible (Figure 3), but it should be noted that the manufacturer recommend using biosensors charged with nickel ions.

### 3.4. The Effect of Enzyme Concentration on Substrate Processing

In order to study whether the change in the signal is proportional to enzyme activity, HIV-1 PR_wt_ was applied in different concentrations, while the Ni-NTA biosensors were loaded with the same amount of His_6_-MBP-VSQNY*PIVQ-mEYFP. Each reaction was measured using the same Ni-NTA biosensor, which was regenerated between measurements.

**Figure 4:**
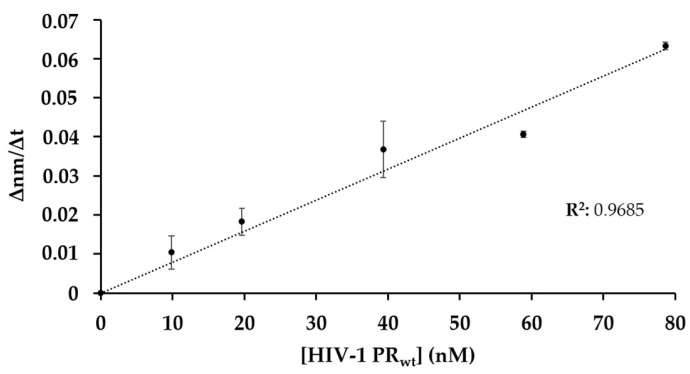
Dependence of initial change in the bio-layer thickness on enzyme concentration. The proteolysis was performed at increasing concentration of HIV-1 PR_wt_. The initial ∆nm/∆t values were determined based on the slopes (2–10 s after initiation of the reaction). Error bars represent SD (n = 2).

The change in the bio-layer thickness over the time (∆nm/∆t) was evaluated to determine the slope of the signal. The initial ∆nm/∆t values were plotted as a function of enzyme concentration (Figure 4) and showed good correlation, indicating that the decrease in the signal is proportional to the enzyme activity, and that the method is suitable for enzymatic measurements.

As the next step, the BLI-based protease assay was applied to investigate the effect of a protease inhibitor on enzyme activity and to study enzyme specificity with different substrates.

### 3.5. Measuring the Effect of an Inhibitor on Protease Activity

To further prove that the change in the optical signal is caused by the proteolysis and not by the spontaneous dissociation of the substrate from the biosensor surface or by non-specific interactions between the immobilized RPFs and the enzyme, the cleavage reactions were performed in the presence of HIV-1 PR inhibitor atazanavir, which has been approved by the Food and Drug Administration (FDA) [43].

**Figure 5:**
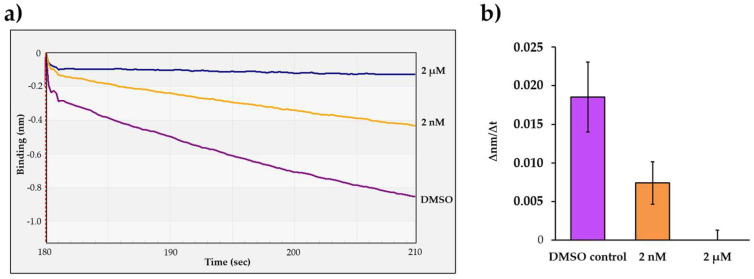
Determination of the inhibitory effect of atazanavir on HIV-1 PR activity. (**a**) Representative sensograms of the enzyme reactions performed by using atazanavir in 2 nM and 2 µM final concentration. (**b**) Comparison of obtained initial ∆nm/∆t values which were determined based on the slopes (2–10 s after initiation of the reaction). Error bars represent SD (n = 2). Representative signal curves are shown for all steps of the assay in Figure S3a.

The His_6_-MBP-VSQNY*PIVQ-mEYFP substrate and HIV-1 PR_wt_ were applied for the cleavage reactions together with atazanavir in 2 nM and 2 µM final concentration. Atazanavir was dissolved in dimethyl sulfoxide (DMSO), and therefore control reactions contained DMSO instead of the inhibitor solution. The most intensive signal change was observed for the control reaction (Figure 5a). As compared to the control, the slope of the signal was lower if atazanavir was applied in 2 nM final concentration, indicating only partial inhibition. In contrast, the change in the BLI signal was only marginal in the presence of 2 µM inhibitor, which implied that the enzyme was completely inhibited at the applied concentration (Figure 5b). Our results further confirmed that the change in the detected signal was caused by substrate proteolysis.

### 3.6. Effect of P2-Leu Substitution in HIV-1 PR MA/CA Cleavage Site

The suitability of the BLI-based protease assay for the comparative analysis of the cleavage efficiencies of different substrates was also tested. The experiments were performed by using His_6_-MBP-VSQNY*PIVQ-mEYFP substrate, which represents the wild-type MA/CA cleavage site of HIV-1 (wt-9res) [15]. A modified version of this substrate was also designed—the P2 residue of the cleavage site was changed to leucine (VSQLY*PIVQ) (mut-9res). The oligopeptide substrate representing the wild-type MA/CA cleavage site was found previously to be a better substrate of HIV-1 PR as compared to the P2-Leu mutant [38,44,45]. Therefore, we expected that the preferential cleavage of the wild-type sequence can be determined by the BLI-based assay. The difference between the cleavage efficiencies was implied by the results of electrophoretic analysis, as well, where the turnover of mut-9res substrate was lower as compared to wt-9res (Figure 1).

**Figure 6:**
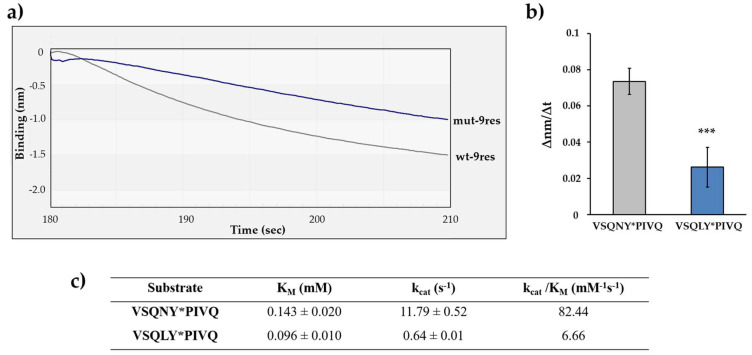
Determination of substrate specificity of HIV-1 PR using P2-modified RFP substrates. (**a**) Representative sensogram of the enzyme reactions with different substrates wt-9res (VSQNY*PIVQ) mut-9res (VSQLY*PIVQ). (**b**) Comparison of obtained ∆nm/∆t values. The initial ∆nm/∆t values were determined based on the slopes (2–10 s after initiation of the reaction). ***: *p* = 0.0004. Error bars represent SD (n = 4). (**c**) Kinetic parameters determined previously for HIV-1 PR using oligopeptide substrates representing 9-residue-long cleavage sites [44]. Representative signal curves are shown for all steps of the assay in Figure S3b.

The cleavage efficiencies of HIV-1 PR_wt_ on the wt-9res and mut-9res RFPs were determined by comparing the initial ∆nm/∆t values (Figure 6a). We found that the decrease in the signal was more intense in the case of wt-9res substrate as compared to mut-9res, and the difference was found to be significant (Figure 6b). This is in agreement with the amino acid preferences determined previously for HIV-1 PR [44,45] and prove preferential cleavage of wild-type MA/CA cleavage site as compared to the P2-Leu mutant (Figure 6c). Our results clearly demonstrated that the real-time BLI-based protease assay may be suitable to study amino acid preferences; therefore, additional substrates were also designed to investigate HIV-1 PR specificity.

### 3.7. Study on the Potential Substrate Groove of HIV-1 PR

It is known that HIV-1 PR recognizes P4-P4′ substrate residues at the active site where the closed conformational flaps cover the bound substrate and are involved in substrate recognition. A study investigating the resistance mutations of HIV-1 PR revealed the existence of substrate binding sites at the enzyme surface distant from the active site [46]. This interaction surface was referred to as the substrate groove and was found to contribute to the binding of P12-P5 and P5′-P12′ substrate residues, making recognition of 24 residues possible. To study the substrate groove, substrates representing a wild-type (DTGNNSQVSQNY*PIVQNLQGQMVH) and a modified (AGSGSGAGSQNY*PIVQAGSGAGSA, the modified positions are underlined) NL4-3 HIV-1 MA/CA cleavage site were designed, and the enzyme–substrate interactions were studied both in silico and in vitro by Laco [46,47]. The mutant HIV-1 MA/CA substrate was found to show weakened intermonomeric interactions with the enzyme, and the processing of the modified substrate was also slower as compared to the wild-type, indicating the contribution of substrate groove binding sites to substrate recognition.

In order to confirm these results in vitro, we designed His_6_-MBP-mApple substrates representing the wild-type and a modified 24-residue-long HIV-1 MA/CA cleavage site sequence (Figure 1b). As compared to the wild-type HIV-1 MA/CA sequence (wt-24res: DTGHSNQVSQNY*PIVQNIQGQMVH), the modified substrate contained the P12-P6 and P5′-P12′ residues of the HIV-1 capsid/p2 cleavage site (mut-24res: GVGGPGHVSQNY*PIVQSQVTNSAT, the modified positions are underlined). The modification was assumed to potentially alter enzyme–substrate interactions at the substrate groove which can be detected by using BLI.

**Figure 7:**
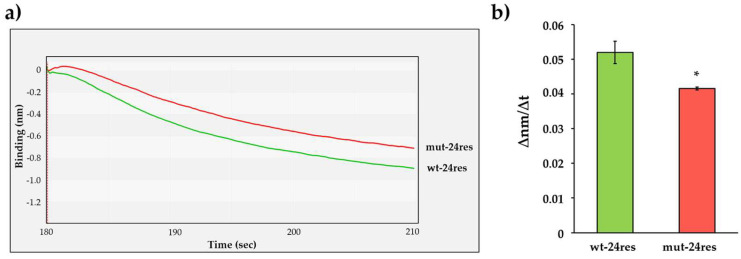
Examination of the substrate groove of HIV-1 PR. (**a**) A representative sensogram of the enzyme reactions performed with wt-24res and mut-24res substrates. (**b**) Comparison of obtained ∆nm/∆t values. The initial ∆nm/∆t values were determined based on the slopes (2–10 s after initiation of the reaction). *: *p* = 0.0463. Error bars represent SD (n = 2). Representative signal curves are shown for all steps of the assay in Figure S3c.

The cleavage efficiencies of wt-24res and mut-24res substrates were determined using the interferometry-based assay (Figure 7a). We detected significantly slower turnover of mut-24res substrate (Figure 7b), which implied preferential cleavage of the wild-type HIV-1 MA/CA site as compared to the mutant, in agreement with the literature data [46]. The differences between the cleavage efficiencies implied that the BLI-based assay is suitable for the investigation of enzyme specificity by using RFPs containing different cleavage sites.

To verify our findings and to exclude the possibility that the obtained differences were caused, e.g., by different quantities of the immobilized substrates, the wt-24res and mut-24res His_6_-MBP-mApple RFPs were used as substrates of HIV-1 PR_wt_ in a magnetic bead-based assay to determine the kinetic parameters (Table 3). The modification of the cleavage site sequence resulted in higher K_M_ and k_cat_ values, while the catalytic constant (k_cat_/K_M_) obtained for the mut-24res substrate was lower as compared to the wt-24res substrate. The results of the BLI- and magnetic bead-based assays were in good agreement and showed preference of HIV-1 PR for the wild-type cleavage site. 

It is important to note that the wild-type and mutant cleavage site sequence may differ in their conformational characteristics. While the cleavage site sequences are located distant from the His_6_ tag, the modifications at this site are expected to have no effect on substrate immobilization, rather may potentially have effect on the structure of the linker between the MBP and the fluorescent tag. We have already observed such difference for RPFs which contained the same wt-9res HIV-1 MA/CA cleavage site sequence (the P12-P12′ sequences were also fully identical) but were fused to different fluorescent proteins: the k_cat_/K_m_ values were almost identical while the individual k_cat_ and K_m_ values were different for mApple and mTurquoise2-fused substrates [13]. The changes in the kinetic parameters (k_cat_ and K_M_) can be explained by the changes in the patterns of enzyme-substrate contacts at least at the enzyme surface (P12-P6 and P6′-P12′), but it remains to be elucidated how sequences and structural features of substrates have effect on substrate binding and product release. Nevertheless, the results obtained for the wt-9res and mut-9res substrates by the gel-based assay (Figure 1C) and by the BLI- (Figure 6b) and oligopeptide-based assays (Figure 6c) are highly comparable and imply higher cleavage rate for the wild-type cleavage site.

### 3.8. BLI Protease Assay Using Non-Purified Substrate

One of the advantages of BLI method is the possibility to use cell lysates for capturing target molecules on the biosensor without any previous time consuming purification steps. The His-tagged proteins that are present in complex biological samples (e.g., in bacterial cell lysates) can be directly used in BLI measurements according to the application notes of the BLItz instrument.

**Figure 8:**
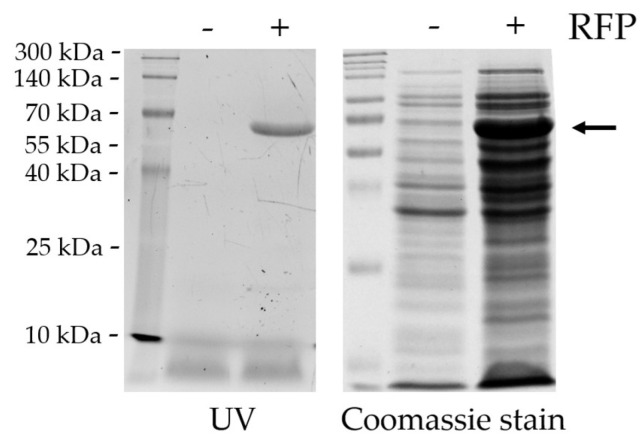
SDS-PAGE analysis of the lysates of non-transformed (RFP−) and transformed (RFP+) BL21(DE3) cells. Arrow shows His_6_-MBP-VSQNYPIVQ-mEYFP protein substrate (~72 kDa) detected in the cell lysates of transformed cells. Before UV transillumination, the proteins were renatured in the gel after separation by reducing SDS-PAGE (using 14% gel), then the gel was stained with Coomassie dye.

We tested whether the RFPs can be captured by the biosensor directly from total lysates of cells. We used the lysates of non-transfected BL21(DE3) cells and those of expressing His_6_-MBP-VSQNY*PIVQ-mEYFP substrate. The cell lysates were analyzed by reducing SDS-PAGE, and the substrate was clearly detectable in the lysates of transformed cells by UV-transillumination (after in-gel renaturation) or by Coomassie staining of the gel, while the RFP was absent from the lysates of non-transformed cells (Figure 8).

**Figure 9:**
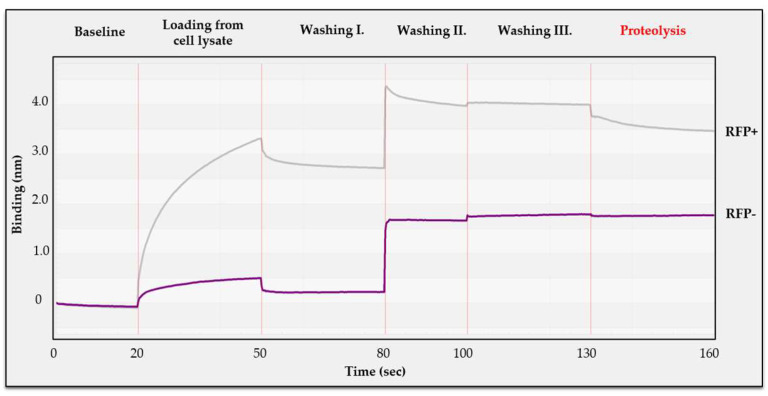
Cleavage reaction performed with non-purified RFP substrate on BLItz instrument. On a Ni-NTA biosensor, a baseline was initially established (tube-mode), then the sensor was merged into the cell lysate either containing (RFP+) or lacking (RFP−) the His_6_-MBP-VSQNY*PIVQ-mEYFP substrate, until saturation were reached (drop-mode). Three individual washing steps were added to remove non-specifically bound proteins (tube-mode). Finally, HIV-1 PR_wt_ was added to the reactions (drop-mode) and the signal change was analyzed.

The experimental setup consisted of the same steps as described above, but an additional tube-mode washing step (Washing step I) was introduced to eliminate non-specifically bound cellular proteins. The contaminants were present in the cell lysates (Figure 8), and the washing steps were expected to remove the molecules non-specifically bound to the Ni-NTA biosensor. The increase in the detected signal between washing steps I and II was caused by switching the lysis buffer to assay buffer, such change was not observed between washing steps II and III. After loading biosensors with the cell lysate of non-transformed cells, only a weak binding signal was detected, which was almost completely diminished by washing (Figure 9). The addition of HIV-1 PR_wt_ did not cause a decrease in the signal, which indicated that none of the non-specifically bound proteins were processed by the enzyme.

In the case of total cell lysate containing the His_6_-MBP-VSQNY*PIVQ-mEYFP substrate, we observed a remarkable binding signal at the loading step, indicating strong affinity binding of His-tagged substrate to the biosensor. The sensogram showed decrease in the signal at the proteolysis step, suggesting a change in the bio-layer thickness due to processing of His_6_-MBP-VSQNY*PIVQ-mEYFP substrate by HIV-1 PR_wt_ (Figure 9).

Our results imply that not only purified but non-purified substrates may also be used for the measurements, as both the purification of the substrates by magnetic beads and their immobilization on biosensors is based on affinity binding of His-tagged proteins to the Ni-NTA surface. Although we have not performed extensive measurements using non-purified RFPs for loading, we assume that total cell lysates may be also used efficiently for substrate immobilization. While most in vitro assays require purification of substrates prior to activity measurements, an advantage of this method may be that the substrates can be loaded onto the sensors directly from the cell lysates, decreasing the time of the experimental procedure.

## 4. Discussion

Due to the significance of proteolytic enzymes in numerous biological processes, in industrial applications (e.g., pharmaceutical and food industry) and in drug development, there is a great need for efficient and sensitive protease assays.

Our research group has developed an His_6_-MBP-FP-based, fluorescent protease assay for HTS-compatible substrate screening, the designed RFP substrate system has been successfully applied to study HIV-1 and TEV PRs [13], Ty1 retrotransposon PR [17], human paternally expressed gene 10 (PEG10) [16], the main protease of the novel severe acute respiratory syndrome coronavirus 2 (SARS-CoV-2) [18] and the non-structural protein 2 protease of the alphavirus Venezuelan equine encephalitis virus (VEEV) [19]. The RFP substrates have versatile applications, can be digested in-solution, can be used in microbead-based assays and the product formation can be detected using either electrophoresis or fluorimetry. Although the primarily designed assay has been successfully applied for the determination of enzymatic kinetic parameters based on the end-point detection, we have not designed such application of the RFP substrates to date, which would enable investigation of protease activity in real time. Therefore, in the present study, we aimed to develop a new experimental approach for the continuous measurement of protease activity.

For this, the BLI method was chosen, which is based on the optical measurement of the thickness of a biomolecular layer on the surface of a biosensor. This method is used mostly to study protein–protein interactions via measuring the increase in the thickness of the bio-layer on the surface sensor caused by intermolecular interactions. We assumed that the BLI technique may be applicable to study proteolysis by following the decrease in the bio-layer thickness in real time. For the enzyme activity measurements, His_6_-MBP-FP substrates were designed, containing the cleavage site of HIV-1 PR. The RFPs were found to be processed by HIV-1 PR_wt_ both in solution and when immobilized to the Ni-NTA biosensor. We proved that the observed changes are not caused by spontaneous substrate dissociation, and the change in the signal was not detected if a catalytically inactive HIV-1 PR_ΔNΔC_ was applied. Our results are in agreement with those of Sharma et al., who used PreScission protease to remove the molecules remaining on the sensor surface and the proteolytic digestion caused the decrease in the binding signal [22]. In that study, the proteolytic step was used to enzymatically remove the proteins from the sensor, and it provided evidence that the measured changes in the signal were caused by intermolecular interactions, rather than by proteolytic activity.

We tested four divalent ions for RFP immobilization and found that Ni^2+^-charged biosensors can be used the most efficiently in the applied experimental system, and we obtained the highest reproducibility for these sensors. The use of regenerated sensors may be advantageous as the lower the number of the applied sensors, the more reduced the costs are. To minimize non-specific binding, blocking agents (e.g., bovine serum albumin, BSA) or non-ionic detergents (e.g., Tween-20) may be applied, the buffer environment can also be further optimized and the chemical cross-linkers can also stabilize the interactions between the Ni-NTA surface and the His-tagged protein. However, it must be considered that blocking proteins (e.g., BSA) may be cleaved by the protease of interest. Additionally, various biosensors are available for immobilization of, e.g., biotinylated, His-tagged and GST-tagged proteins; therefore, a wide variety of protein substrates can be adapted for the BLI-based protease assay. Antibody-coated biosensors are also available to capture His-tagged proteins, but it needs to be considered that these antibodies may be potentially cleaved by the protease of interest. Thus, it is recommended to use such sensors that are not susceptible to proteolysis.

Our results showed that the initial rate of signal decrease is proportional to the enzyme activity, and the developed method can be used to detect the effect of a protease inhibitor on HIV-1 PR activity. Both experiments verified that the decrease in the signal at the proteolysis step is a consequence of the digestion of the bio-layer but not by the spontaneous dissociation of the immobilized substrate. This observation was further confirmed by using inactive HIV-1 PR_ΔN__ΔC_ enzyme. The BLI has already been applied to study the interaction of HIV-1 PR inhibitors with DNA damage-inducible 1 (Ddi1) retroviral-like protease of *Leishmania major* [48]. The interferometry was used in this study to determine binding affinity but not to directly detect the effect of inhibitors on enzyme activity. Our results imply that the BLI-based protease assay may be suitable to detect the effect of protease inhibitors; however, detailed inhibitory studies were out of the scope of this work.

To test whether the designed method is suitable for the determination of amino acid preferences, we prepared RFPs representing the wild-type MA/CA cleavage site [13], and a His_6_-MBP-VSQLY*PIVQ-mEYFP substrate containing a cleavage site mutated at the P2 position was also prepared. The cleavage efficiencies were determined using the BLI-based assay, and the obtained differences were compared to those determined for synthetic oligopeptide substrates [38,44,45]. The comparison showed good correlation of the results, and showed a lower initial rate of substrate processing for the P2-Leu mutant substrate, for which lower catalytic efficiency was determined as compared to the wild-type. Our results revealed that the BLI-based method using the His_6_-MBP-FP substrate system can be used for the determination of relative cleavage efficiencies in a real-time assay, which, until now, was possible only in end-point reactions.

The BLI-based assay was applied to investigate the substrate groove surface-binding sites of HIV-1 PR, which are responsible for the recognition of P5-P12 and P5′-P12′substrate residues. The existence of the extended binding sites at the surface of HIV-1 PR was verified in vitro as the enzyme showed differential cleavage efficiency of wild-type and modified substrates [46]. The contributions of S5 site to substrate recognition have already been justified experimentally for human T-cell leukemia virus type 1 (HTLV-1) [49] and for HTLV-2 and HTLV-3 PRs [50]. Furthermore, a similar strategy was applied to study the substrate groove of yeast Ty1 retrotransposon protease [17], but the existence of substrate groove binding sites in HIV-1 PR [46] was not confirmed experimentally by independent studies to date. We designed His_6_-MBP-mEYFP substrates representing the 24-residue-long, wild-type HIV-1 MA/CA cleavage site and its variant, which was modified at P12-P6 and P5′-P12′ positions. The relative initial rates measured by the developed BLI assay were higher for the wild-type RFP, which is in agreement with the higher catalytic efficiency determined for this substrate using the microbead-based fluorometric assay. Our results provide experimental evidence for the existence of surface binding sites of HIV-1 PR, and imply that the relative cleavage efficiencies can be determined real-time using BLI. A series of modified substrates may be designed in the future to study the surface residues in substrate recognition in details.

While the BLI is based on the immobilization of purified protein onto the biosensor surface, we have tested whether non-purified RFPs can be applied to prepare the biomolecular layer. We successfully applied the lysates of cells expressing the His_6_-MBP-VSQNY*PIVQ-mEYFP, which was found to be susceptible to proteolysis after immobilization. The application of non-purified substrates may decrease experimental time and costs, but it is important to note that the primary purification of substrates may be advantageous, as the elimination of numerous contaminants may increase loading efficiency and help to avoid interference with non-specifically bound proteins.

The main advantages and limitations of the BLI-based protease assay are described as follows.

During the measurements, the optimal temperature of enzyme reactions must be considered. The temperature optimum of HIV-1 PR is close to 37 °C, but our measurements were performed at slightly suboptimal temperature (~25 °C) because the applied BLItz instrument has no temperature control. While the initial rate of signal change may be higher at the temperature optimum of the applied enzyme, the use of OctetRed96e instrument (Pall Forté Bio, Fremont, CA, USA) may be desired, as this instrument can control temperature between 15 °C to 40 °C. However, our results demonstrate that the enzymatic studies may be performed at slightly suboptimal temperature in case of HIV-1 PR. Another advantage of OctetRed96e over BLItz is that the use of a multi-channel system may enable simultaneous measurements with multiple substrates, and can be adapted to 96-well microtiter plates. The automated measurements make this method suitable for high-throughput analyses and they can decrease the time of analyses.

Depending on the type of the applied biosensor, the buffer conditions that are optimal for both immobilization and enzymatic catalysis need to be considered. In our experiments, the proteolysis step was performed at lower pH (~5.5), but the effect of relatively lower pH on substrate immobilization was counteracted by the limited dissociation in the drop-mode measurement; the slow spontaneous dissociation can be decreased by applying higher pH (>6.0).

To determine enzyme kinetic parameters, the use of the microbead-based assay would be preferred, as the preparation of reaction mixtures in microcentrifuge tubes or in microtiter plate can ensure setting proper substrate concentrations. In the BLI-based assay, a substrate concentration gradient may be hard to set. For this, a non-cleavable protein would be used for the dilution of the RFP substrate solution, but we assume that the ratio of the cleavable substrate and non-cleavable proteins (e.g., His_6_-MBP-FP and His_6_-MBP, respectively) actually immobilized to sensor surface would not be quantified reliably.

An advantage of BLI is that this is a label-free method, the change in the signal is caused directly by the proteolysis of the substrate, and the product formation is detected by optical measurement. Accordingly, there is no need for fluorescent readout, and the stability of fluorescent protein does not limit this method. In the herein described work, we applied His_6_-MBP-FP substrates. The product formation was detected by the optical measurements, but the BLI-based analyses may be completed by affinity bead- and PAGE-based fluorometric measurements, as well. Consequently, the same His_6_-MBP-FP recombinant substrates might be applied in multiple measurements with different readouts. Another advantage of the use of recombinant substrates in the BLI-based assay is that the proteins can be prepared easily and in a cost-efficient manner by using bacterial expression systems [13]. In contrast, the synthesis of the labelled or unlabeled oligopeptides, used in HPLC- and FRET-based techniques, is more expensive and time-consuming. Another advantage of the method is that there is no need for extensive use of organic solvents, as in the case of HPLC-based separation of the oligopeptides after their cleavage reaction [45].

In conclusion, the herein described BLI-based protease assay is a method that can be used to follow proteolysis in real-time by continuous optical measurement. While the number of real-time homogenous (e.g., FRET-based) and heterogeneous (e.g., SPR) protease assays is limited [24], the herein described BLI-based approach may be a good alternative for continuous measurement of product formation. This technique is a new approach for the use of the His_6_-MBP-FP recombinant protein substrate system developed in our laboratory. Due to the flexibility of the substrates, the BLI-based assay may be adapted to several different substrates and proteases.

We demonstrated that this methodology is useful to study multiple substrate binding sites of HIV-1 PR and confirmed the existence of substrate groove in HIV-1 PR.

## Figures and Tables

**Table 1:** The oligonucleotide primers coding for cleavage sites of HIV-1 PR. “F” and “R” indicate forward and reverse primers, respectively. Cleavage site sequences are also indicated, asterisks indicate cleavage position. The primer coding for the wt-9res cleavage site was described previously [13].

Cleavage Site Name and Sequence	Primer Sequence
**wt-9res** VSQNY*PIVQ	F: 5′-TAAAGTGAGCCAGAACTATCCGATTGTGCAGG-3′
	R: 5′-CTAGCCTGCACAATCGGATAGTTCTGGCTCACTTTAAT-3′
**mut-9res** VSQLY*PIVQ	F: 5′-TAAAGTGAGCCAGCTGTATCCGATTGTGCAGG-3′
	R: 5′-CTAGCCTGCACAATCGGATACAGCTGGCTCACTTTAAT-3′
**wt-24res** DTGHSNQVSQNY*PIVQNIQGQMVH	F: 5′-TAAAGATACCGGCCATAGCAACCAGGTGAGCCAGAACTATCCGATTGTGCAGAACATTCAGGGCCAGATGGTGCATG-3′
	R: 5′-CTAGCATGCACCATCTGGCCCTGAATGTTCTGCACAATCGGATAGTTCTGGCTCACCTGGTTGCTATGGCCGGTATCTTTAAT–3′
**mut-24res** GVGGPGHVSQNY*PIVQSQVTNSAT	F: 5′-TAAAGGCGTGGGCGGCCCGGGCCATGTGAGCCAGAACTATCCGATTGTGCAGAACCAGGTGACCAACAGCGCAACCG-3′
	R: 5′-CTAGCGGTTGCGCTGTTGGTCACCTGGTTCTGCACAATCGGATAGTTCTGGCTCACATGGCCCGGGCCGCCCACGCCTTTAAT–3′

**Table 2:** Parameters determined for different divalent cations. The corresponding sensograms are shown in Figure 3. Values are shown for two independent measurements.

Ion	Sample	ka (1/Ms)*10^3^	kd (1/s)×10^3^	R_equilibrium_	R_max_	R^2^
nickel	1	0.37 ± 0.12	0.42 ± 0.014	0.93	2.49	0.98
	2	0.47 ± 0.11	0.45 ± 0.012	1.08	2.54	0.98
cobalt	1	1.08 ± 0.15	1.19 ± 0.020	0.61	1.56	0.98
	2	2.59 ± 0.15	0.69 ± 0.012	0.81	1.12	0.98
copper	1	0.29 ± 0.14	1.85 ± 0.033	0.27	2.82	0.97
	2	0.36 ± 0.12	1.55 ± 0.060	0.45	3.26	0.97
zinc	1	1.55 ± 0.12	0.81 ± 0.013	1.34	2.36	0.98
	2	0.32 ± 0.14	0.93 ± 0.021	0.68	3.53	0.97

**Table 3:** Kinetic parameters determined for HIV-1 PR. For the measurements, the His_6_-MBP-mApple substrates representing wild-type and modified 24-residue-long cleavage sites were applied. The modified residues of HIV-1 MA/CA cleavage site are underlined. ^1^ For comparison, values determined previously for wt-9res substrate are also shown [13].

Substrate	K_M_ (mM)×10^−3^	k_cat_ (s^−1^)×10^−3^	k_cat_/K_M_ (mM^−1^s^−1^)
VSQNY*PIVQ ^1^	2.7 ± 0.2	50 ± 0.8	18.52 ± 1.40
DTGHSNQVSQNY*PIVQNIQGQMVH	1.68 ± 0.928	26.3 ± 1.464	15.67 ± 8.71
GVGGPGHVSQNY*PIVQSQVTNSAT	7.68 ± 0.003	40.4 ± 0.005	5.26 ± 0.02

## Data Availability

The data presented in this study are available on request from the corresponding author.

## Supplementary Materials

The following are available online at https://www.mdpi.com/article/10.3390/v13061183/s1. Figure S1: SDS-PAGE analysis of uncleaved RFP substrates and cleavage products after proteolysis with HIV-1 PR_wt_. Figure S2: Cleavage of His_6_-MBP-VSQNY*PIVQ-mEYFP substrate with HIV-1 PR_wt_ and trypsin. Figure S3: Representative sensograms showing signal curves from baseline to proteolysis steps.
