# Supplementary material for: Development of a Bio-Layer Interferometry-Based Protease Assay Using HIV-1 Protease as a Model
Source: Viruses. 2021 Jun 21;13(6):1183. doi: 10.3390/v13061183 (PMC8235736; doi:10.3390/v13061183)

Figure S3. Representative sensograms showing signal curves from baseline to proteolysis steps. **a**) Effect of atazanavir on the activity of HIV-1 PR_{wt}. **b**) Cleavage of wt-9res (grey) and mut-9res (blue) substrates with HIV-1 PR_{wt}. **c**) Cleavage of wt-24res (red) and mut-24res (purple) substrates with HIV-1 PR_{wt}.

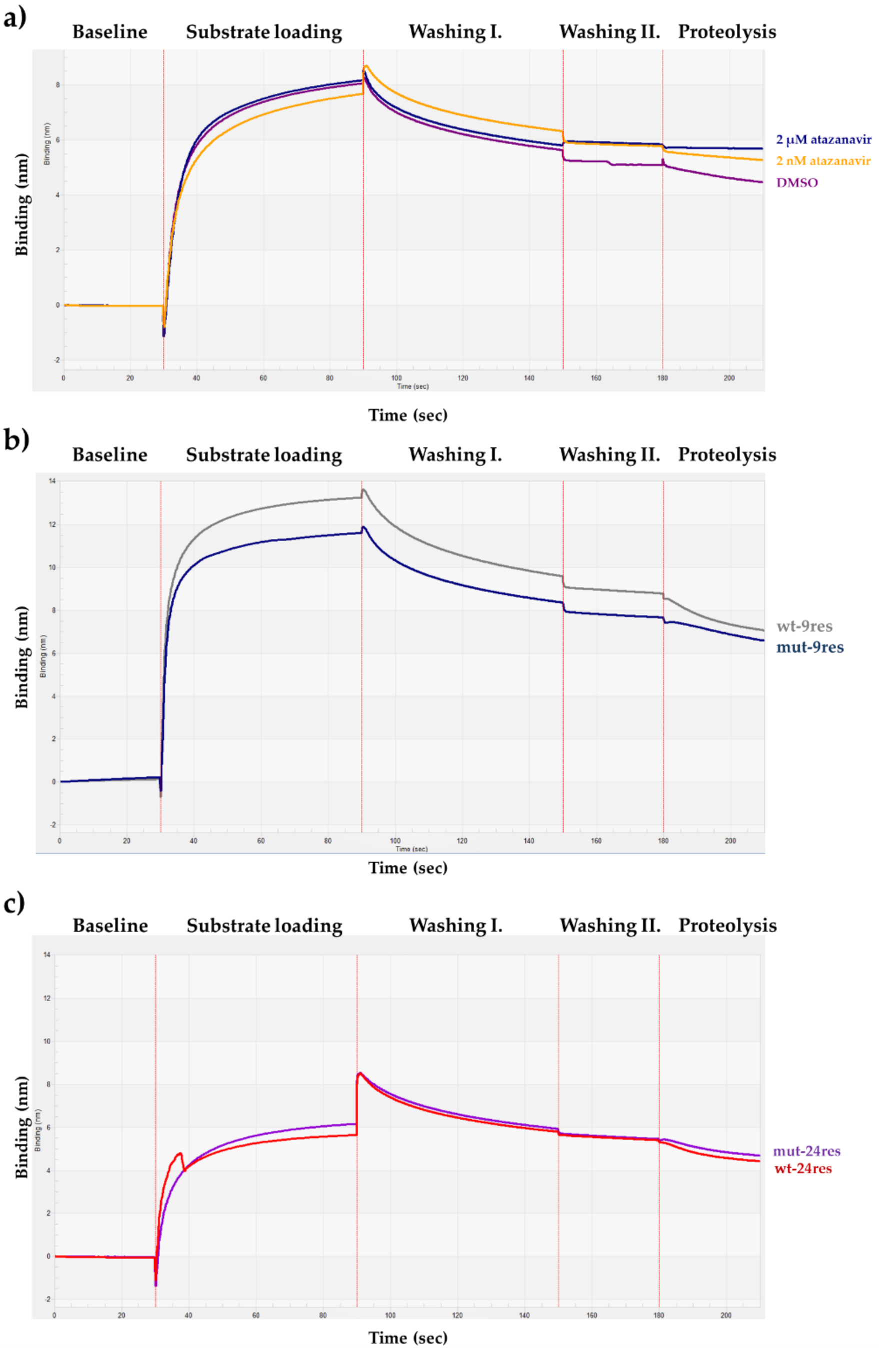

Supplement: Supplementary file 1 [file viruses-13-01183-s001.zip › Figure_S3.pdf]
