# Supplementary material for: Development of a Bio-Layer Interferometry-Based Protease Assay Using HIV-1 Protease as a Model
Source: Viruses. 2021 Jun 21;13(6):1183. doi: 10.3390/v13061183 (PMC8235736; doi:10.3390/v13061183)

Figure S2. Cleavage of His_6-MBP-VSQNY*PIVQ-mEYFP substrate with HIV-1 PR_{wt} and trypsin. The sensorgram is shown from baseline to proteolysis steps. In proteolysis step, HIV-1 PR_{wt} (purple) and trypsin (red) were applied, while the control reaction was absent from HIV-1 PR and contained only buffer (yellow).

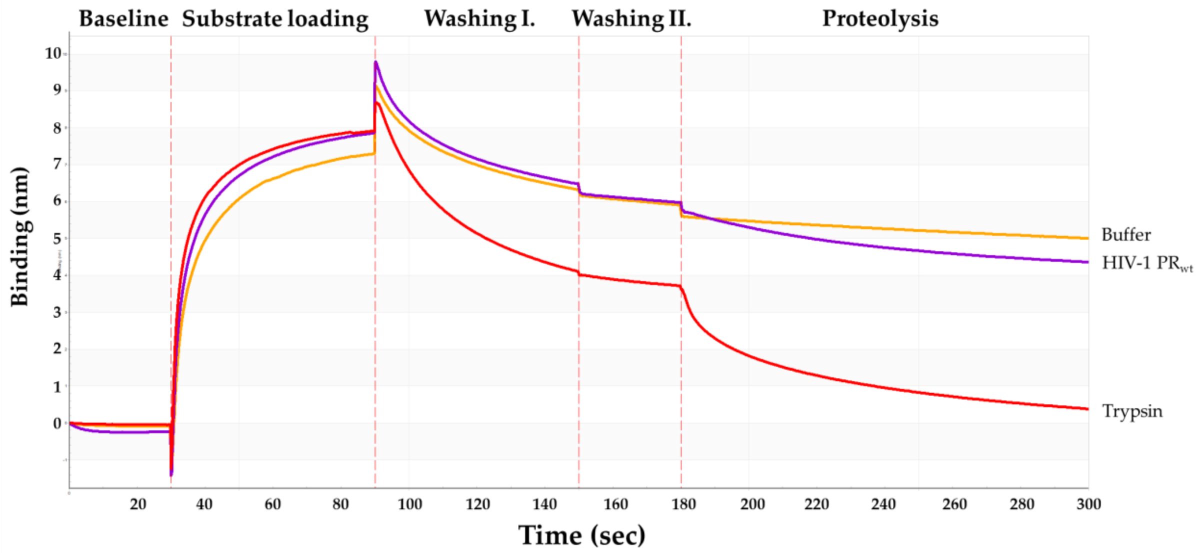

Supplement: Supplementary file 1 [file viruses-13-01183-s001.zip › Figure_S2.pdf]
