# Supplementary material for: Development of a Bio-Layer Interferometry-Based Protease Assay Using HIV-1 Protease as a Model
Source: Viruses. 2021 Jun 21;13(6):1183. doi: 10.3390/v13061183 (PMC8235736; doi:10.3390/v13061183)

Figure S1. SDS-PAGE analysis of uncleaved RFP substrates and cleavage products after proteolysis with HIV-1 PR_{wt}. The proteins were visualised in the representative gel using Coomassie staining. The arrow shows the full-length substrates, the N- and C-terminal cleavage products are indicated with dashed arrow and asterisks, respectively. The C-terminal cleavage products - containing the fluorescent protein - which are released from 9res and 24res substrates differ in their molecular weight.

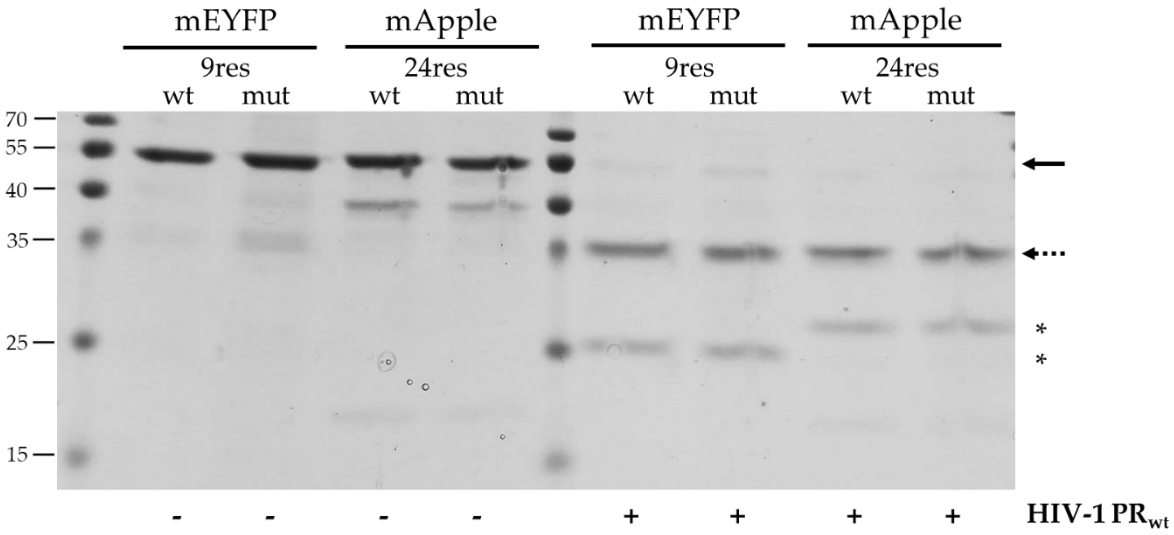

Supplement: Supplementary file 1 [file viruses-13-01183-s001.zip › Figure_S1.pdf]
